# Aiweixin, a Traditional Uyghur Medicinal Formula, Extends the Lifespan of* Caenorhabditis elegans*

**DOI:** 10.1155/2019/3684601

**Published:** 2019-01-13

**Authors:** Binggen Zhu, Kayama Jo, Ping Yang, Jurat Tohti, Jian Fei, Kaisaier Abudukerim

**Affiliations:** ^1^College of Xinjiang Uyghur Medicine, Hetian, Xinjiang 848000, China; ^2^Pudong New Area Mental Health Center, Tongji University School of Medicine, Shanghai 200092, China; ^3^Shanghai Research Center for Model Organisms, Shanghai 201203, China; ^4^Tongji University School of Life Sciences and Technology, 200092, China

## Abstract

Aiweixin (AWX) is a traditional Uyghur medicine prescription, which has been used to treat senile diseases for a long time. We investigate whether the AWX extends the lifespan of* Caenorhabditis elegans*. The AWX decoction was the conventional product for clinical use. The wild-type* Caenorhabditis elegans* (N2) and mutational worms,* daf-16(mu86), glp-1(e2141), daf-2(e1370), *and* eat-2(ad465), *were applied for the lifespan analysis. We found that the lifespan of the N2 adults' worm received 0.005 and 0.01 volume of AWX/total volume was extended significantly, compared to the control without treatment of AWX. The AWX at 0.01 volume of AWX/total volume significantly prolonged the life of both mutational worms,* daf-16 (mu86)* and* eat-2(ad465)*, but did not increase the lifespan of the mutational worms,* daf-2(e1370)* and* glp-1(e2141)*. These results indicated that the AWX significantly extended the lifespan of wild-type nematodes, and the life extension effect of AWX was related to the germline longevity pathway and IIS signaling pathway but independent of DAF-16/FOXO.

## 1. Background

The nematode* Caenorhabditis elegans* is a popular model organism used in biomedical fields, because of its transparent body, short life span, ease of maintenance and genetic manipulation, and amenability to high-throughput screening [1]. The worm possesses genes homologous to two-thirds of those involved in human disease and the signaling pathways controlling the aging process are conserved between* Caenorhabditis elegans* and humans [2–4]. These features make it an ideal model for aging research and assessing compounds that can increase stress resistance and/or even promote healthy aging in humans [4–7]. Increasing studies disclosed that some of Chinese medicinal herbs and most of secondary plant compounds influence ageing, stress resistance, and distinct signaling pathways in the nematodes [5–8].

Aiweixin (AWX) is a traditional Uyghur medicine prescription, which has been used to treat heart diseases, neuropsychiatric disorders (such as depression) and senile diseases for a long time [9–12]. AWX consists of 15 ingredients including* Dracocephalum moldavicum *L.,* Eletteria cardamomum *(L.) Maton,* Salix caprea* L. (Salicaceae) flowers,* Lavandula augustifolia *(lavender),* Borago officinalis* L. (Boraginaceae) stems and leaves,* Borago officinalis* L. (Boraginaceae) flower,* Nardostachys jatamansi* DC. root and rhizome (Nardostachyos Radix et Rhizoma),* Bombyx mori *(Abresham) silk cocoons,* Usnea longissima* Ach.,* Rosa rugosa* Thunb. flowers,* Syzygium aromaticum* L.,* Lindera caudata* (Nees) Hook.f.,* Myristica fragrans* (Houtt.), and* Crocus sativus* L. and Moschus [8]. The HPLC fingerprint of AWX showed 4 large peaks, by using HPLC–UV-ESI-TOF-MS system. Peaks 1-4 contain chebulic acid, gallic acid, protocatechuic acid, and eugenol, respectively [13].

Previous experiments demonstrated that therapeutic effects of AWX in a rat model of myocardial ischemia reperfusion injury were possibly via alleviation of oxidative stress [14, 15], and the antioxidative defense mechanisms were involved in the protective action of AWX against the toxicity of chromium (VI) in* Caenorhabditis elegans* [13]. The aim of this work was to investigate the lifespan-extending effects of AWX and study the possible mechanisms by which AWX delays the aging process in* Caenorhabditis elegans*. For this purpose, wild-type (N2) and several mutational worms, such as* daf-16 (mu86), glp-1(e2141), daf-2 (e1370), *and* eat-2 (ad465), *were used in our studies. These mutational lines would help us to search several known signaling pathways which were involved in the aging of nematodes [4].

## 2. Methods

### 2.1. Preparation of AWX

The AWX decoction was the conventional product for clinical use, manufactured by Xinwei Pharmaceutical Factory (Hetian, Xinjiang Uygur Autonomous Region, PR China). According to the Pharmacopeia of PR China [7], the product is made from* Dracocephalum moldavicum *L. (15 g),* Eletteria cardamomum *(L.) Maton (15 g),* Salix caprea* L. (Salicaceae) flowers (10 g),* Lavandula augustifolia *(lavender) (15 g),* Borago officinalis* L. (Boraginaceae) stems and leaves (10 g),* Borago officinalis* L. (Boraginaceae) flower (10 g),* Nardostachys jatamansi* DC. root and rhizome (Nardostachyos Radix et Rhizoma) (10 g),* Bombyx mori *(Abresham) silk cocoons (50 g),* Usnea longissima* Ach. (3 g),* Rosa rugosa* Thunb. flowers (15 g),* Syzygium aromaticum* L. (15 g),* Lindera caudata* (Nees) Hook.f. (10 g),* Myristica fragrans* (Houtt.) (15 g),* Crocus sativus* L. (0.6 g), and Moschus (0.2 g). The AWX decoction was prepared as described elsewhere [13].

### 2.2. *Caenorhabditis elegans* Strain and Maintenance

The wild-type* Caenorhabditis elegans* strain N2 (Bristol) and mutational worms,* daf-16 (mu86), glp-1(e2141), daf-2 (e1370), *and* eat-2 (ad465),* were provided by the* Caenorhabditis* Genetics Center (University of Minnesota, Minneapolis, MN). Nematodes were generally incubated at 20°C on nematode growth media (NGM) plates with* E. coli* OP50. For culture of* daf-2 (e1370)* and its wild-type control, nematodes were firstly developed at 16°C for 3 days and then transferred to 20°C for the desired stage of development.

### 2.3. Lifespan Analysis

The lifespan assay was performed basically as described elsewhere [16]. Age-synchronized day 1 adult N2 and mutational nematodes were transferred to a 96-well plate with 1-2 worms in 80*μ*L of S-complete liquid medium containing various AWX drug concentrations (0.001, 0.005, 0.01, 0.05 volume of AWX/total volume, respectively) in each well.* E. coli* OP50 and FUDR were added to the medium. Therefore, AWX treatment was applied throughout the whole life of the worms since day 1. Plates were sealed with tape (Nunc) to prevent evaporation. Then the temperature was shifted from 20°C to 25°C. Survival was assessed every day or every other day until death using the touch-provoked method. The fraction of animals alive was scored using a microscope on the basis of movement. The nematodes were considered dead when they failed to respond to touch using a platinum loop. The lifespan assay was repeated in three independent trails.

### 2.4. Statistical Analyses

GraphPad Prism 6.01 was used for statistical analyses. For the lifespan assay, Kaplan-Meier survival analysis was conducted, and p values were calculated using the log-rank (Mantel-Cox) test. Values of* P* < 0.05 were considered significant.

## 3. Result

### 3.1. The Effect of AWX on the Lifespan of Wild Type* Caenorhabditis elegans*

**Figure 1:**
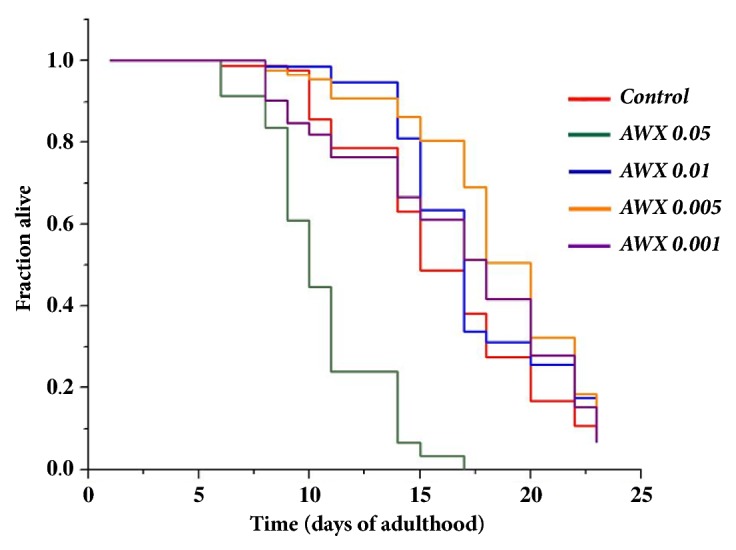
Effects of AWX on the lifespan of wild-type N2 nematodes. The data were presented in Table 1. The lifespan of the N2 adults' worm received AWX at 0.005 and 0.01 was extended significantly, compared to the control without treatment of AWX (p<0.0001 and p=0.0091, respectively). Whereas the lifespan of worms received AWX at 0.05 was significantly lower than the one of the control without treatment of AWX (p<0.0001). All worms were dead at 17 day of experiment with the treatment of 0.05 AWX, while some worms were still alive at 23 day of experiment in other groups.

As shown in Figure 1 & Table 1, the lifespan of the N2 adults' worm received AWX at 0.005 (18.296 ± 0.422) and 0.01 (17.130 ± 0.414) was extended significantly, compared to the control without treatment of AWX (15.506 ± 0.447, p<0.0001 and p=0.0091, respectively). The increasing percentages of AWX at 0.005 and 0.01, compared to the control, were 17.99% and 10.47%, respectively. The effect of AWX was seen since 11 day and was basically in a dose-dependent manner. AWX at 0.001 did not extend significantly the lifespan of* Caenorhabditis elegans* compared to the control (16.333 ± 0.621 vs 15.506 ± 0.447, and p=0.2726), whereas the lifespan of worms rthat eceived AWX at 0.05 was significantly lower than the one of the control without treatment of AWX (10.565 ± 0.277 vs 15.506 ± 0.447, p<0.0001). All worms were dead at 17 day of experiment with the treatment of 0.05 AWX, while some worms were still alive at 23 day of experiment in other groups (Figure 1 & Table 1). Therefore, AWX at high doses was quite harmful for the survival of nematodes.

### 3.2. The Lifespan-Extending Effects of AWX Was DAF-2 and GLP-1 Dependent, but EAT-2 and DAF-16 Independent

As shown in Table 2, the lifespan-extending effects of AWX were not seen in the mutational worms,* daf-2(e1370)* and* glp-1(e2141)*. The lifespan of* daf-2(e1370)* and* glp-1(e2141) *received AWX at 0.01 was not significantly different from the one in control without treatment of AWX (35.34 ± 1.200 vs 33.88 ± 0.9682, p=0.2696; 18.30 ± 0.2490 vs 18.26 ± 0.3969, p=0.8184). AWX at 0.01 slightly increased the lifespan of the mutational worms,* daf-2(e1370)*, but the effect did not reach significant level. Therefore, the lifespan-extending effects of AWX should be DAF-2 and GLP-1 dependent.

However, the lifespan of* daf-16 (mu86)* and* eat-2(ad465) *received AWX at 0.01 was significantly higher than the one in control without treatment of AWX (16.09 ± 0.3302 vs 12.67 ± 0.2814, p<0.0001; 33.31 ± 0.5561 vs 28.36 ± 0.5138, p<0.0001). The increasing percentages with the treatment of AWX at 0.01, compared to the control, were 26.99% and 17.45%, respectively. AWX at 0.01 significantly prolonged the life of both mutational worms,* daf-16 (mu86)* and* eat-2(ad465)*. In the consequence of results, the lifespan-extending effects of AWX were independent of both DAF-16/FOXO and EAT-2.

## 4. Discussion

Recently, Zhao et al. identify two forms of death in ageing* Caenorhabditis elegans* [17]. The first form, “P death” which showed severe swelling of the posterior pharyngeal bulb, is associated with pharyngeal invasion and proliferation of bacteria and mainly occurred in mid adulthood (at mean age of 12 days). The antibiotic, Carbenicillin, that was used to treat bacteria which were food for worms, significantly reduced the P death in midlife [17]. According to the phytochemical analysis of AWX by high-performance liquid chromatography-diode array detection (HPLC-DAD), the eugenol and gallic acid were identified in the second and third high peaks of AWX, respectively [13]. The eugenol attenuated the virulence of enterohemorrhagic* Escherichia coli *O157:H7 (EHEC) [18]. Eugenol at 0.005% prolonged* Caenorhabditis elegans* survival in the presence of EHEC. The gallic acid increased the longevity of worm only in the presence of live bacteria, but not dead bacteria, depending on its antibacterial capacities [19]. Therefore, it is not impossible that the decline of P death by the eugenol and gallic acid in the AWX might be one of mechanisms which promote the survival of nematodes. Moreover, researchers also found that eugenol at high doses exhibited nematicidal activity in the* Caenorhabditis elegans* model [20, 21], which may explain the toxic effect of high concentration of AWX for the worms in our previous [13] and present experiments.

Another form of death in ageing* Caenorhabditis elegans* is the “p death,” which showed marked atrophy of the posterior pharyngeal bulb, presented an exponential increase in mid-to-late life (at mean age of 22 days) [17]. We found that AWX prolonged the lifespan of* eat-2(ad465) *mutant, although the lifespan already extended in* eat-2 *mutant compared to the wild type. The lifespan extension observed in the* eat-2* mutant was largely attributable to reduction in the frequency of P death, according to the studies of Zhao et al. [17]. Hence, it is possible that AWX might not only protect worms from bacteria invasion and reduce the P death, but also exerted genuine anti-aging effects and lessened the p death, thus leading to that the* eat-2* mutant lived much longer at the appropriate doses of AWX. On the other hand, Zhao et al. found that the increase lifespan of* Caenorhabditis elegans* in germline defective mutant* glp-1* was, to a large extent, attributed to delaying of “p death” [17]. Our studies showed the lifespan extension effect of AWX was removed in the* glp-1(e2141)* mutant, highlighting that holding off “p death” should be the important route by which AWX promote the survival of nematodes. It is noteworthy that the* glp-1*mutant that induces germline depletion enhances the resistance of worms to pathogenic bacteria [22]. The effect was recently found to be involved in the activation an innate immunity related gene, named irg-7, which might be a novel mediator of longevity in germlineless animals [23].

The most unintentional finding in our study was that AWX exposures led to significantly prolonged lifespan in* daf-16 (mu86) *mutants, but not in* daf-2(e1370)* mutants, indicating that the described AWX effects did not depend on DAF-16/FOXO activity, although it was in a DAF-2/insulin/IGF-1 receptor-dependent manner, to some extent. So far, three most important downstream lifespan-regulatory transcription factors of insulin/IGF-1 signaling (IIS) pathway in* Caenorhabditis elegans* that have been identified are DAF-16/FOXO, heat shock transcription factor 1 (HSF-1), and SKN-1/nuclear factor erythroid 2 (NRF2) [24, 25]. Therefore, HSF-1 and/or SKN-1 might be responsible for the effect of AWX. Besides DAF-16/FOXO, HSF-1, or SKN-1 also are essential for the longevity of animals with reduced IIS [24, 25]. HSF-1 binds to specific regions of DNA containing heat shock elements, and triggers the induction of genes encoding molecular chaperones, such as HSP-70 and HSP-16, whose overexpression extends lifespan [26]. Thus, HSF-1 appears to lead to longevity by upregulating the chaperone network that enhances the proper folding of various proteins [23, 24]. Over expression of constitutively nuclear SKN-1 extends lifespan in a DAF-16/FOXO-independent manner [27]. SKN-1, an oxidative stress-responsive NRF transcription factor, mediates the expression of genes involved in detoxification and stress responses, and promotes protein homeostasis through regulating proteasome production, which contributes to a longer lifespan [23, 24, 26]. Meanwhile, in addition to DAF-16/FOXO, other pathways, such as steroidal hormone signaling and innate immune response, may play an important role in the lifespan-extending effects of germline removal. Reduction-of-function mutations in the gene* daf-12*, which encodes a nuclear hormone receptor (NHR), or in genes like* daf-9*, which encode proteins that synthesize sterol ligands for DAF-12, prevent loss of the germline from extending lifespan [28, 29].

Future lifespan assays with various* Caenorhabditis elegans* mutants and RNAi strains as well as gene expression profile via RT-PCR will reveal whether or not the HSF-1, SKN-1, DAF-12, DAF-9, and IRG-7 pathway are responsible for the observed AWX effects. Moreover, complete quantitative phytochemical studies of the AWX, measuring pharyngeal pumping rate and longitudinal pathology analysis on individual worms and using dead bacteria previously treated by antibiotics as food for worms, would be helpful to seek mechanisms of the lifespan extension effect of AWX.

## 5. Conclusion

In conclusion, the AWX significantly extended the lifespan of wild-type nematodes. Further studies on several* Caenorhabditis elegans* mutants indicated that the lifespan extension effect of AWX was related to the germline longevity pathway and IIS signaling pathway but independent of DAF-16/FOXO.

## Figures and Tables

**Table 1:** The data analysis of effects of AWX on the lifespan of wild-type N2 nematodes.

Treatment	Lifespan (days)	Percentage of control	^#^Total number of animals Died/Total	*∗p* value
Control	15.506 ± 0.447	-	77/84	-
AWX 0.05	10.565 ± 0.277	-31.86%	92/92	< 0.0001
AWX 0.01	17.130 ± 0.414	10.47%	69/74	0.0091
AWX 0.005	18.296 ± 0.422	17.99%	81/87	< 0.0001
AWX 0.001	16.333 ± 0.621	5.33%	66/72	0.2726

^#^Total number of animals was the number of wild-type N2 nematodes used in the beginning of experiment. Died/Total was the number of dead worms in the end of experiment vs the total number of worms used in the study (which was equal to the number of worms in the beginning of experiment).

*∗p* value, compared to control (without treatment of AWX).

**Table 2:** The data analysis of effects of AWX on the lifespan of mutational worms, *daf-2(e1370)*, *glp-1(e2141), daf-16 (mu86)*, and* eat-2(ad465).*

Strain & Treatment	Lifespan (days)	Percentage of control	^#^Number of animals	*∗p* value
*daf-2 (e1370)* -control	33.88 ± 0.9682	-	40	-
*daf-2 (e1370)* 0.01	35.34 ± 1.200	4.33%	38	0.2696

*glp-1 (e2144)* -control	18.26 ± 0.3969	-	62	-
*glp-1 (e2144)* 0.01	18.30 ± 0.2490	0.25%	66	0.8184

*daf-16 (mu86)* -control	12.67 ± 0.2814	-	48	-
*daf-16 (mu86)* 0.01	16.09 ± 0.3302	26.99%	47	< 0.0001

*eat-2 (ad465)*- control	28.36 ± 0.5138	-	61	-
*eat-2 (ad465)* 0.01	33.31 ± 0.5561	17.45%	58	< 0.0001

^#^Number of animals means the number of worms used in the experiments.

*∗p* value, compared to respective controls (without treatment of AWX at 0.01).

## Data Availability

The data (in the form of PZFX file) used to support the findings of this study are available from the corresponding author upon request. GraphPad Prism 6.01 was used for statistical analyses.
